# Sex‐specific elevated incidence of glaucoma associated with topiramate versus valproate or lamotrigine in epilepsy, not migraine: A population‐based cohort study

**DOI:** 10.1002/epi.70087

**Published:** 2026-01-10

**Authors:** Cuiling Wei, Rachel Yui Ki Chu, Rachel Lancey Lai, Florinda Hui‐Ning Chu, Ian Yu Hin Leung, William Chun Yin Leung, Franco Wing Tak Cheng, Thomas Chi Ho Lam, Ian Chi Kei Wong, Esther Wai Yin Chan, Francisco Tsz Tsun Lai

**Affiliations:** ^1^ Centre for Safe Medication Practice and Research, Department of Pharmacology and Pharmacy, Li Ka Shing Faculty of Medicine The University of Hong Kong Hong Kong SAR China; ^2^ Department of Obstetrics and Gynaecology The Chinese University of Hong Kong & Prince of Wales Hospital Hong Kong SAR China; ^3^ Division of Neurology, Department of Medicine, Queen Mary Hospital University of Hong Kong Hong Kong SAR China; ^4^ Pharmacy Department Gleneagles Hong Kong Hospital Hong Kong SAR China; ^5^ Department of Pharmacy The University of Hong Kong‐Shenzhen Hospital Shenzhen China; ^6^ Hong Kong Eye Hospital Hong Kong SAR China; ^7^ Department of Ophthalmology and Visual Sciences The Chinese University of Hong Kong Hong Kong SAR China; ^8^ Aston School of Pharmacy Aston University Birmingham UK; ^9^ Laboratory of Data Discovery for Health (D^2^4H) Hong Kong Science Park Hong Kong SAR China; ^10^ The University of Hong Kong Shenzhen Institute of Research and Innovation Shenzhen China; ^11^ Department of Family Medicine and Primary Care, School of Clinical Medicine, Li Ka Shing Faculty of Medicine The University of Hong Kong Hong Kong SAR China

**Keywords:** glaucoma, lamotrigine, topiramate, valproate

## Abstract

**Objective:**

Topiramate has been linked to increased glaucoma risk, potentially through mechanisms involving ocular fluid shifts. However, comparative risks vs other antiseizure medications (ASMs) and variation by sex or indication remain uncertain. This study evaluates glaucoma incidence in topiramate initiators compared to valproate or lamotrigine users among patients with epilepsy or migraine.

**Methods:**

We conducted a retrospective active‐comparator, new‐user cohort study using electronic health records from the IQVIA Medical Research Data among patients with epilepsy or migraine initiating topiramate, valproate, or lamotrigine. Patients with prior ASM use, limited washout period and follow‐up, or pre‐existing glaucoma were excluded. The outcome was incident glaucoma within 1 year, censored at glaucoma occurrence, death, discontinuation, switch, or September 30, 2023. Covariates included age, sex, race, lifestyle factors, comorbidities, and medication history. Propensity score–based inverse probability weighting balanced characteristics, and crude and weighted Cox models estimated hazard ratios (HRs) with 95% confidence intervals (CIs). Subgroup analyses were conducted by sex, age, and indication.

**Results:**

The cohort included 688 topiramate, 4490 valproate, and 4179 lamotrigine initiators. After weighting, the 1‐year absolute risk increase was approximately 2.4% when comparing topiramate to valproate, and about 2.0% compared to lamotrigine. Topiramate was associated with higher glaucoma risk vs valproate (adjusted HR 2.66, 95% CI 1.12–6.32) and lamotrigine (adjusted HR 3.57, 95% CI 1.76–7.26). Risks were elevated in female (vs valproate: HR 5.31, 95% CI 1.48–19.08; vs lamotrigine: HR 5.73, 95% CI 2.38–13.79) or epilepsy patients (vs valproate: HR 2.23, 95% CI 1.04–4.76; vs lamotrigine: HR 5.08, 95% CI 2.32–11.14), but not in male or migraine patients.

**Significance:**

Topiramate use substantially increases glaucoma risk compared with valproate and lamotrigine, particularly among female or epilepsy patients. No significant association in male or migraine patients was observed. These findings may inform targeted ophthalmologic monitoring in high‐risk groups and use of alternative ASMs.

**Key points**

- Topiramate use was linked to a significantly higher glaucoma risk than valproate or lamotrigine, especially early after initiation.
- Elevated risk was concentrated in women and epilepsy patients; no significant increase was seen in men or migraine patients.
- Higher topiramate doses, more common in epilepsy, may partly explain the elevated glaucoma risk observed.
- Sex‐ and indication‐specific risk stratification may guide safer ASM selection and targeted ophthalmologic monitoring.

## 1 INTRODUCTION

Glaucoma is a leading cause of irreversible blindness, marked by progressive optic neuropathy that results in visual field loss.^1^ Among various risk factors for glaucoma, the use of topiramate^2^ has received increased attention due to its association with acute angle‐closure glaucoma and related ocular complications.^3^ Topiramate, an antiseizure medication (ASM) first approved by the U.S. Food and Drug Administration (FDA) in 1996 for epilepsy and migraine, exerts its antiseizure effects through multiple biological mechanisms including modulation of voltage‐gated sodium and calcium channels and inhibition of carbonic anhydrase isoenzyme and enhancement of γ‐aminobutyric acid (GABA)–mediated neurotransmission.^4^ It is believed to induce an increased risk of glaucoma through mechanisms involving supraciliary or ciliochoroidal effusions that rotate the ciliary body anteriorly and displace the iris‐lens diaphragm forward, leading to anterior chamber shallowing, narrowing of the iridocorneal angle, and often an acute myopic shift.^2^, ^5^ Case reports and case series have documented instances of acute angle‐closure glaucoma, myopic shifts, and choroidal effusions after or during topiramate use, with patients often experiencing symptoms like blurred vision and eye pain shortly after starting therapy.^6^, ^7^

This link between topiramate and glaucoma risk is supported by accruing real‐world observational studies. A cohort study from Taiwan found that topiramate users experienced more than seven times the risk of glaucoma compared to non‐users within the first month after drug initiation.^8^ Similarly, case–control studies from the United States and Canada observed elevated glaucoma risks among topiramate users, with modest increases of around 20% for current users and 50% for new users, and, notably, a more than fivefold increase in patients younger than 50.^9^, ^10^ A disproportionality analysis based on the FDA Adverse Event Reporting System (FAERS) in 2024 also provided supporting data on this association.^11^

These studies are not without limitations, however. Of note, most of these studies are case–control or cohort studies that compare topiramate users to “non‐users,” which are susceptible to indication bias by not accounting for the potential impact from the neurological conditions per se, rather than topiramate. In fact, patients with epilepsy or migraine are typically prescribed some ASM rather than none. Patients who are not prescribed any ASM are likely milder cases or may be receiving other unrecorded therapies. The magnitude of the association observed in previous studies may have been overestimated as a result. Indication bias aside, these studies confer limited clinical implications for prescription decisions across viable drug options because “non‐use” is oftentimes not a realistic option.

To address this gap, we conducted an active‐comparator, new‐user cohort study among patients with epilepsy or migraine to examine the risk of glaucoma among topiramate users compared to valproate and lamotrigine users. We selected valproate, an older‐generation ASM, as one comparator because it is the most prescribed broad‐spectrum ASM for all seizure types and is endorsed in certain migraine prophylaxis guidelines.^12^, ^13^ Similarly, lamotrigine, a newer‐generation ASM, was chosen as another active comparator for its overlapping indications with topiramate including focal and generalized seizures and increasing off‐label use for migraine.^14^ We hypothesized that topiramate would be associated with a higher glaucoma risk compared to valproate and lamotrigine among patients with epilepsy or migraine.

## 2 METHODS

### 2.1 Data source

The IQVIA Medical Research Data (IMRD) is a comprehensive repository of non‐identified electronic patient health records collected from UK General Practitioner (GP) clinical systems, specifically incorporating data supplied by practices using EMIS Health Web system. This EMIS‐sourced data captures longitudinal information on 5.98 million patients from more than 191 GP practices in England. The database includes detailed primary care records such as patient demographics, symptoms, diagnoses, prescriptions, immunizations, laboratory tests, and lifestyle data. Its strengths lie in providing high‐quality, real‐world, validated data that are broadly representative of the UK population, enabling efficient research on rare exposures and outcomes in the field of pharmacoepidemiology.^15^

### 2.2 Cohort selection

We conducted two retrospective active‐comparator, new‐user cohort studies using electronic health record data: one comparing new users of topiramate and valproate, and another comparing new users of topiramate and lamotrigine. For each drug group (topiramate, valproate, and lamotrigine), we identified new users as individuals with at least two prescriptions of the respective drugs, ensuring a minimum of 365 days of clinic registration prior to the first prescription to establish a washout period and minimize misclassification of ongoing users. The index date was set as the first prescription date of the initiating drug. In each study, we combined the topiramate group with the respective comparator group (valproate or lamotrigine) and further refined each cohort by excluding individuals with prior use of other antiseizure medications on or before the index date, and those with less than 90 days of potential follow‐up until the study end date (September 30, 2023). In addition, we excluded participants with a history of glaucoma‐related diagnoses or procedures (identified using Read Codes version 2 and the International Classification of Diseases, Tenth Revision, Clinical Modification [ICD‐10‐CM], detailed in Table S1), or anti‐glaucoma medications (identified using generic name including bimatoprost, tafluprost, latanoprost, travoprost, timolol, apraclonidine, brimonidine, dorzolamide, methazolamide, and acetazolamide) prior to the index date. Indication for study drug use was identified based on the first recorded diagnosis of epilepsy or migraine occurring on or before the date of the first ASM prescription (diagnosis codes detailed in Table S1). The final cohorts were restricted to individuals with epilepsy or migraine.

### 2.3 Exposures

Exposure was defined as initiation of topiramate compared to either valproate or lamotrigine as the primary ASM in the respective cohorts. Drug prescriptions were identified under the Anatomical Therapeutic Chemical (ATC) classification prefix ”N03A” (antiepileptics) and specific generic names: topiramate, valproic acid, sodium valproate and lamotrigine. Exposure groups were assigned based on the drug with the earlier first prescription date in each cohort.

### 2.4 Outcomes and follow‐up

The primary outcome was the incidence of glaucoma, defined by the first occurrence of a glaucoma‐related diagnosis, procedure, or prescription for glaucoma‐specific medications after the index date. Glaucoma incidence included diagnoses such as primary open‐angle glaucoma, angle‐closure glaucoma, and related conditions, as well as procedures like laser trabeculoplasty, trabeculectomy, and anterior chamber interventions, as detailed in Table S1. Follow‐up began on the index date and continued for up to 1 year or until the earliest of glaucoma outcome, death, drug discontinuation, switch to another ASM, or study end (September 30, 2023). Drug discontinuation was defined as no subsequent prescription after the estimated end date of last prescription supply, calculated as the issue date plus 30 days if drug quantity was missing or plus the actual quantity in days otherwise.

### 2.5 Covariates

Baseline covariates were assessed at or prior to the index date and included demographic factors (age, sex, race), lifestyle factors, comorbidities, and concurrent medication use. Age was calculated as the difference between the year of the index date and the year of birth. Race was categorized into groups such as White, Black, Asian, and Unknown/Other, as defined in the database. Lifestyle factors included smoking status and alcoholism. Comorbidities were identified using diagnostic codes recorded before or on the index date, including diabetes, hypertension, bipolar disorder, and cataract. For diabetes and hypertension, diagnosis codes were supplemented with evidence of antidiabetic or antihypertensive prescriptions to enhance ascertainment. Medication use within 1 year prior to the index date was evaluated for steroids, anticholinergics, antihistamines, and antidepressants. All covariates were binary (present/absent) unless otherwise specified. Detailed diagnosis codes and medication codes for lifestyle factors, comorbidities, and concurrent medications are provided in Table S1.

These covariates were selected based on their established associations with glaucoma risk, potential to confound treatment assignment, or relevance to ASM indications for epilepsy and migraine. Demographic factors such as age, sex, and race were included as fundamental confounders, since younger age and certain racial groups like Asian individuals are known to increase glaucoma susceptibility.^9^, ^16^ Lifestyle factors like smoking and alcoholism were chosen for their links to elevated intraocular pressure, which may contribute to glaucoma development.^17^, ^18^ Comorbidities including diabetes and hypertension were prioritized as major modifiable risk factors because diabetes is associated with increased risk through mechanisms like optic nerve damage and neovascularization,^19^ whereas hypertension can impair ocular blood flow and elevate intraocular pressure.^20^ Bipolar disorder was added to minimize indication bias due to its overlap with ASM prescribing, and cataract was incorporated as a common ocular condition influencing eye‐related outcomes. Concurrent medications such as steroids, anticholinergics, antihistamines, and antidepressants were evaluated to control for therapies that might independently raise intraocular pressure, trigger angle‐closure glaucoma, or cause ocular side effects interacting with study drugs.^11^

### 2.6 Statistical analysis

Baseline characteristics were summarized using descriptive statistics, stratified by treatment initiator (topiramate vs comparator). Continuous variables were presented as means with standard deviations (SDs) or medians with interquartile ranges (IQRs), as appropriate, whereas categorical variables were reported as frequencies and percentages. Standardized mean differences (SMDs) were calculated to assess balance between groups, with an SMD threshold of less than .1 indicating adequate balance.

Propensity scores (PSs) were estimated using multivariable logistic regression, with treatment initiator (topiramate vs comparator) as the outcome and baseline covariates as predictors, including sex, age, race, diabetes, hypertension, bipolar disorder, alcoholism, smoking status, cataract, steroid use, anticholinergic use, antihistamine use, antidepressant use, and indication. Stable inverse probability of treatment weights (IPTWs) was derived from the PSs to balance the covariates between groups.

Crude and IPTW‐weighted Cox proportional hazards models were employed to estimate hazard ratios (HRs) with 95% confidence intervals (CIs). The proportional hazards assumption was verified using Schoenfeld residuals. One‐year absolute risk differences were calculated from event‐free probability at 365 days, derived from Kaplan–Meier estimates for crude and weighted survival analyses.

Subgroup analyses were performed by stratifying the cohort on sex (female, male), age (<18, 18–49, ≥50 years), and indication (epilepsy, migraine). For each subgroup, PS models were refitted (excluding the stratifying variable), IPTW weights recalculated, and Cox models re‐estimated to obtain subgroup‐specific absolute risk differences and HRs. Sensitivity analyses assessed robustness: (1) incorporating Fine–Gray model to account for death as a competing event in the survival analysis; (2) extending the washout period to 500 days or shortening it to 180 days to evaluate the impact of new‐user classification; (3) redefining indication categories using all diagnoses of epilepsy and migraine recorded prior to the first ASM prescription, classifying patients as epilepsy‐only, epilepsy‐migraine (those with both diagnoses), or migraine‐only to evaluate potential heterogeneity among overlapping indication groups; and (4) relaxing the requirement of at least two prescriptions and re‐running the analyses including patients with only one single prescription of the study drug to assess the potential exclusion of individuals who may develop early glaucoma shortly after treatment initiation.

Post hoc analyses were subsequently conducted based on observations from the main results. First, to explore a possible dose‐level relationship between topiramate and glaucoma, we calculated the mean daily dose of topiramate by dividing the cumulative dose during the observation period by the follow‐up duration for each topiramate user. Two dose‐restricted analyses were then performed by excluding the lowest 25% and 50% of topiramate users based on mean daily dose, and comparing the remaining topiramate users to the full cohorts of valproate and lamotrigine users. Second, to assess the interaction effect of sex and indication, we tested the significance of sex and indication interaction term in a multivariable Cox model and conducted stratified crude and subgroup‐specific IPTW‐weighted Cox analyses across four subgroups (women with epilepsy, women with migraine, men with epilepsy and men with migraine). Third, motivated by the observed sex‐specific differences in the main analysis, we added a binary covariate for exogenous estrogen use within one year prior to the index date into the propensity score model and re‐estimated the IPTW‐weighted Cox models. Using this updated model, we conducted stratified analyses by sex, by age groups among females (<18, 18–49, ≥50 years), and by estrogen use.

All analyses were performed using R version 4.2.0. Two‐sided *p‐*values < .05 were considered statistically significant.

## 3 RESULTS

**FIGURE 1:**
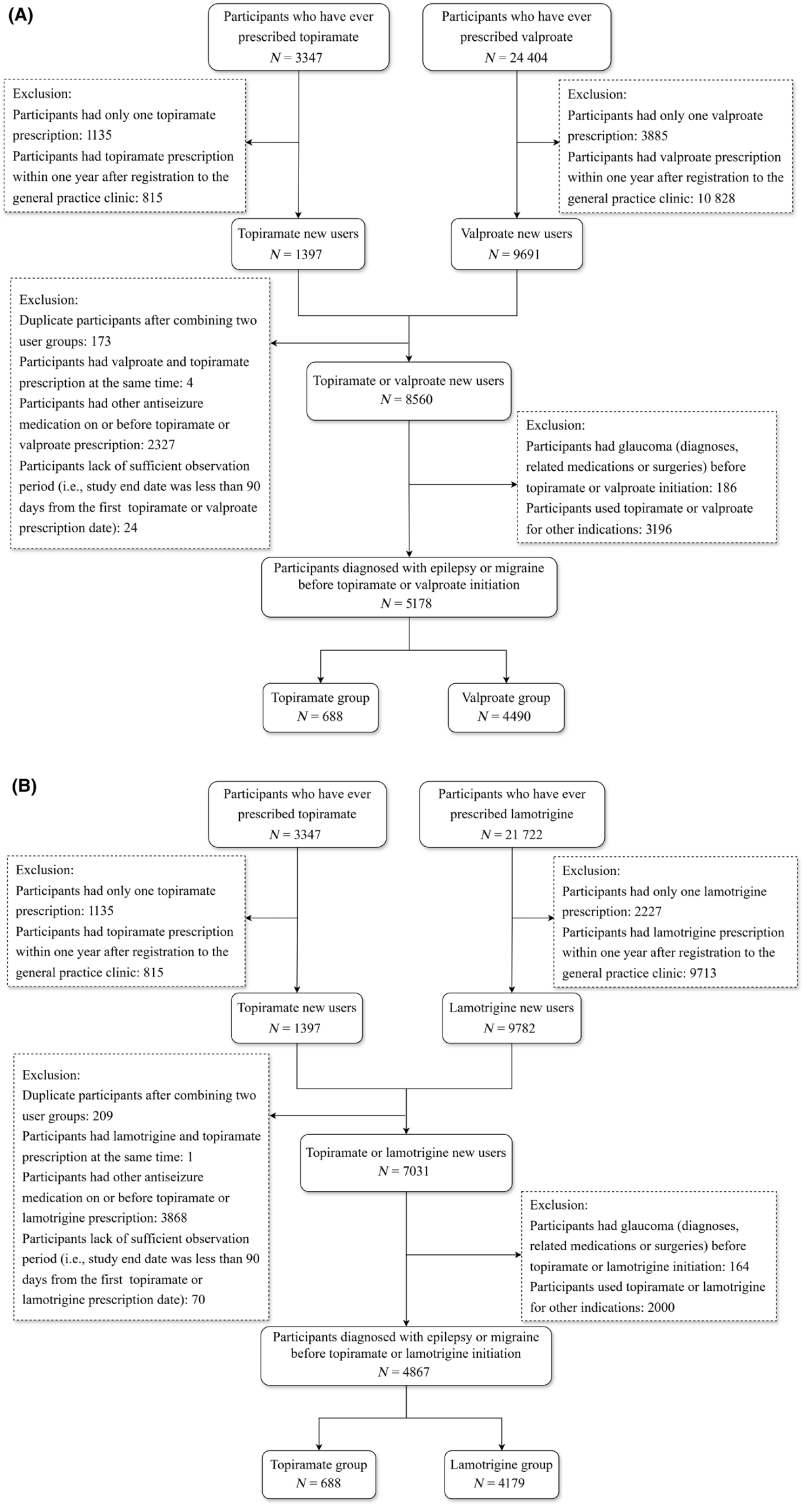
(A) Topiramate and valproate user cohort selection. (B) Topiramate and lamotrigine user cohort selection.

The cohort selection flowcharts were shown in Figure 1A,B. We identified, respectively, 688, 4490, and 4179 topiramate, valproate, and lamotrigine new users. Compared to both comparator groups, topiramate users were younger (mean age 38.2 vs 43.8 years for valproate; 38.5 vs 41.7 years for lamotrigine), more likely to be female (75.6% vs 48.5% for valproate; 75.7% vs 61.5% for lamotrigine), and more commonly prescribed for migraine rather than epilepsy (73.7% vs 21.1% for valproate; 75.3% vs 24.2% for lamotrigine), as shown in Table 1. Topiramate users had lower proportions of patients with baseline alcoholism compared to both groups (6.5% vs 6.0% for valproate; 6.7% vs 12.4% for lamotrigine) and lower smoking rates compared to lamotrigine users, but higher rates compared to valproate users (19.2% vs 15.7% for valproate; 19.6% vs 25.1% for lamotrigine). As for baseline comorbidity, diabetes prevalence was lower among topiramate users in both comparisons (1.8% vs 4.0% for valproate; 1.7% vs 2.9% for lamotrigine), as was hypertension (5.3% vs 9.7% for valproate; 5.5% vs 8.6% for lamotrigine) and cataract (2.0% vs 5.1% for valproate; 2.0% vs. 3.8% for lamotrigine). Bipolar disorder prevalence was higher among topiramate users compared to valproate users (25.6% vs 18.2% for valproate) but similar to lamotrigine users (25.7% vs 27.1% for lamotrigine). As for medication use within 1 year prior to baseline, antidepressant use was notably higher among topiramate users in both comparisons (37.5% vs 19.9% for valproate; 37.8% vs 24.1% for lamotrigine). Substantial imbalances in patient characteristics were observed between groups before IPTW weighting but achieved balance after weighting, with all SMDs <.1 as demonstrated in Figure S1A,B.

**TABLE 1:** Characteristics of topiramate, valproate, and topiramate new users.

	Topiramate new users, *N* = 688	*Topiramate* vs *valproate*			*Topiramate* vs *lamotrigine*		
		Valproate new users, *N* = 4490	*p* value^a^	SMD^b^	Lamotrigine new users, *N* = 4179	*p* value^a^	SMD^b^
Age, years, mean, (SD)^c^	38.53 (16.59)	43.78 (26.47)	<.001	.237	41.65 (20.43)	<.001	.167
Female, *N* (%)	521 (75.7)	2174 (48.4)	<.001	.587	2571 (61.5)	<.001	.31
Race, *N* (%)			<.001	.324		<.001	.184
White	321 (46.7)	1496 (33.3)	–	–	1807 (43.2)	–	–
Asian or Asian British	48 (7.0)	200 (4.5)	–	–	152 (3.6)	–	–
Black	10 (1.5)	98 (2.2)	–	–	84 (2.0)	–	–
Unknown/Other	309 (44.9)	2696 (60.0)	–	–	2136 (51.1)	–	–
ASM indication, *N* (%)			<.001	1.296		<.001	1.187
Epilepsy	170 (24.7)	3550 (79.1)	–	–	3166 (75.8)	–	–
Migraine	518 (75.3)	940 (20.9)	–	–	1013 (24.2)	–	–
Alcoholism at baseline, *N* (%)	46 (6.7)	268 (6.0)	.441	.029	517 (12.4)	<.001	.195
Smoke, *N* (%)	135 (19.6)	706 (15.7)	.012	.102	1048 (25.1)	.002	.131
Diabetes at baseline, *N* (%)	12 (1.7)	182 (4.1)	.002	.138	123 (2.9)	.08	.079
Hypertension at baseline, *N* (%)	38 (5.5)	436 (9.7)	<.001	.158	358 (8.6)	.007	.119
Cataract at baseline, *N* (%)	14 (2.0)	229 (5.1)	<.001	.166	159 (3.8)	.019	.105
Bipolar disorders at baseline, *N* (%)	177 (25.7)	816 (18.2)	<.001	.183	1132 (27.1)	.486	.031
Anticholinergic use at baseline, *N* (%)	5 (.7)	102 (2.3)	.006	.127	58 (1.4)	.201	.065
Antihistamine use at baseline, *N* (%)	23 (3.3)	190 (4.2)	.304	.047	146 (3.5)	.911	.008
Antidepressant use at baseline, *N* (%)	260 (37.8)	888 (19.8)	<.001	.406	1009 (24.1)	<.001	.298
Follow‐up time, median, [IQR]^d^	281.00 [132.00, 365.00]	365.00 [252.00, 365.00]	–	–	365.00 [320.50, 365.00]	–	–

^a^ For continuous variables, the *p*‐value was derived from a one‐way test. For categorical variables, the *p*‐value was obtained using the Fisher's exact test.

^b^ Standard mean difference.

^c^ Standard deviation.

^d^ Interquartile range, the range between the first quartile (Q1) and third quartile (Q3).

### 3.1 Topiramate vs valproate

**FIGURE 2:**
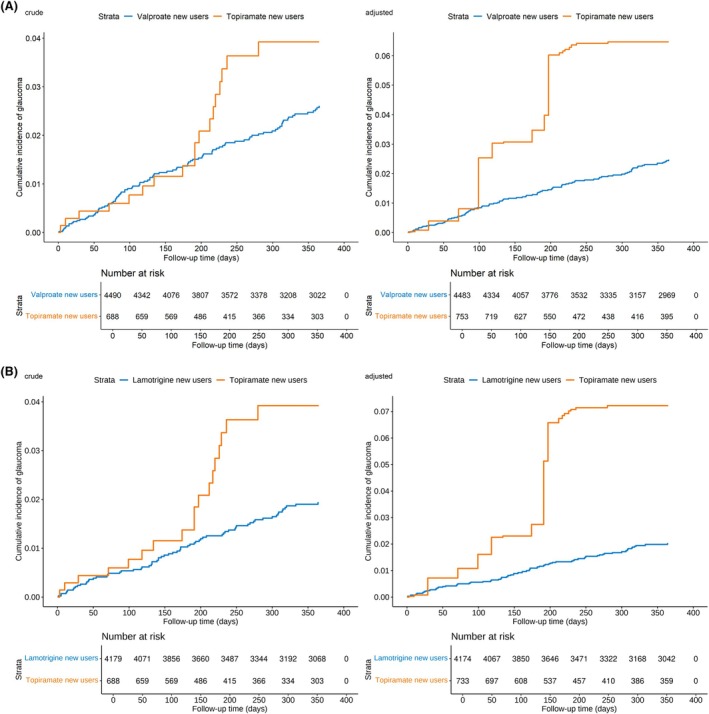
(A) Crude and adjusted cumulative incidence of glaucoma among topiramate and valproate users. (B) Crude and adjusted cumulative incidence of glaucoma among topiramate and lamotrigine users.

**FIGURE 3:**
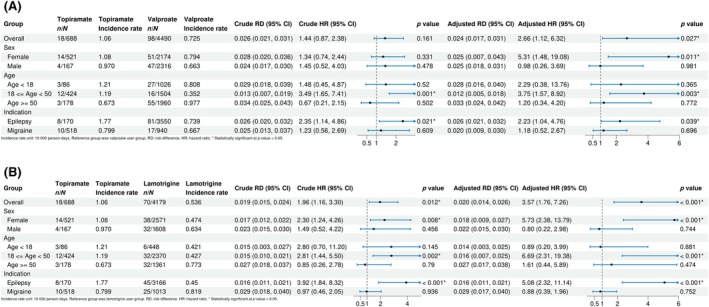
(A) Forest plot of main and subgroup results (topiramate and valproate cohort). (B) Forest plot of main and subgroup results (topiramate and lamotrigine cohort).

Figure 2A shows the cumulative glaucoma risk curves. The divergence of two groups occurred at around Day 200 in the crude analysis and intensifying after IPTW weighting, where topiramate users displayed steep increases beginning at Day 100 and again at Day 200. In the topiramate vs valproate comparison shown in Figure 3A, we observed 18 events among 688 topiramate users (incidence rate 1.060 per 10 000 person‐days) compared to 98 events among 4490 valproate users (incidence rate .725 per 10 000 person‐days). Median follow‐up time was shorter for topiramate users than that of valproate user (289 days [IQR 133–365] vs 365 days [IQR 252–365] for valproate). The crude risk difference of topiramate group over valproate group was .026 (95% CI .021–.031) and crude HR was 1.44 (95% CI .87–2.38). After IPTW adjustment, topiramate users experienced a 166% higher risk of glaucoma compared to valproate users (adjusted HR [aHR] 2.66, 95% CI 1.12–6.32), with risk difference of .024 (95% CI .017–.031).

Subgroup analyses revealed notable sex differences. Female patients taking topiramate had an over fivefold higher glaucoma risk than those on valproate after adjustment (aHR 5.31, 95% CI 1.48–19.08). In contrast, male patients showed no significant difference (aHR .98, 95% CI .26–3.69). In age‐based subgroups, the glaucoma risk was concentrated in the adults aged 18‐49, exhibiting a 3.75 times higher hazard (aHR 3.75, 95% CI 1.57–8.92). Pediatric patients showed no statistically significant difference (aHR 2.29, 95% CI .38–13.76).

When examined by indication, we found that patients with epilepsy receiving topiramate had a significantly increased risk of glaucoma compared with those receiving valproate (aHR 2.23, 95% CI 1.04–4.76). No statistically significant difference was observed for migraine patients (aHR 1.18, 95% CI .52–2.67). Among epilepsy patients, the average daily dose of topiramate was 87.5 mg/day, whereas in migraine patients it was 68.3 mg/day. The difference was statistically significant (*p‐*value of *T*‐test < .001), indicating that epilepsy patients generally received higher daily doses of topiramate than migraine patients.

### 3.2 Topiramate vs lamotrigine

The cumulative glaucoma risk curves in Figure 2B demonstrated that topiramate users had a sharp risk increase in cumulative glaucoma incidence compared to lamotrigine users at around 200 days after drug initiation in both crude and IPTW‐weighted analyses. We observed 70 events among 4179 lamotrigine users (incidence rate .536 per 10 000 person‐days, Figure 3B). Median follow‐up time was shorter for topiramate users than for lamotrigine users (289 days [IQR 133–365] vs 365 days [IQR 321–365] for lamotrigine). The crude risk difference was .019 (95% CI .015–.024) and crude HR was 1.96 (95% CI 1.16–3.30). The IPTW‐adjusted risk difference was .020 (95% CI .014–.026) and aHR was 3.57 (95% CI 1.76–7.26), demonstrating a statistically significant increased risk of glaucoma associated with topiramate use.

Among female patients, topiramate showed a significantly increased risk of glaucoma after adjustment (aHR 5.73, 95% CI 2.38–13.79). Male patients, however, showed no significant difference (aHR .80, 95% CI .22–2.98). Age‐stratified analyses indicated that patients 18–49 years of age experienced 569% higher risk with topiramate vs lamotrigine (aHR 6.69, 95% CI 2.31–19.38), whereas those under 18 years (aHR .89, 95% CI .20–3.99) and equal to or above 50 years (aHR 1.61, 95% CI .44–5.69) showed comparable risks.

Indication‐specific analyses revealed that patients with epilepsy receiving topiramate faced over 5 times higher glaucoma risk than those receiving lamotrigine (aHR 5.08, 95% CI 2.32–11.14), whereas no significant difference was observed for migraine patients (aHR .88, 95% CI .39–1.96).

### 3.3 Sensitivity analysis

**FIGURE 4:**
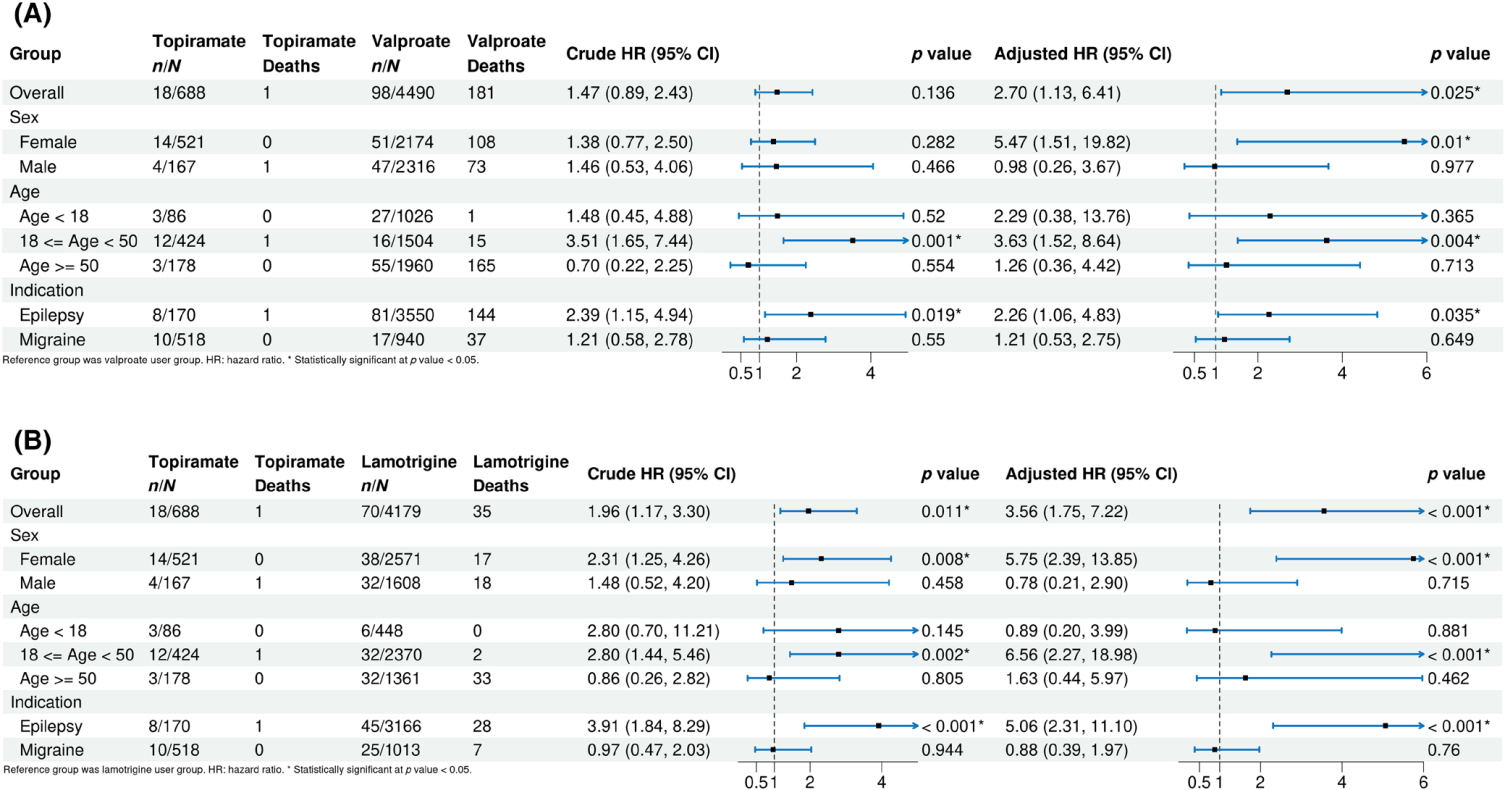
(A) Forest plot of Fine–Gray model results (topiramate and valproate cohort). (B) Forest plot of Fine–Gray model results (topiramate and lamotrigine cohort).

Sensitivity analyses using the Fine–Gray model (Figure 4A,B), which accounts for competing risks from death, yielded results that were consistent with the main Cox regression analyses. The overall aHRs remained statistically significant for both comparisons (topiramate vs valproate: aHR 2.70, 95% CI 1.13–6.41; topiramate vs lamotrigine: aHR 3.56, 95% CI 1.75–7.22), confirming the robustness of our primary findings. Moreover, analyses using different washout periods (180 and 500 days) demonstrated robust results consistent with the primary analysis across subgroups (Figures S2A,B and S3A,B). The overall aHRs remained statistically significant across both washout periods for topiramate vs valproate (180‐day washout: aHR 2.47, 95% CI 1.06–5.71; 500‐day washout: aHR 2.73, 95% CI 1.14–6.55) and lamotrigine (180‐day washout: aHR 3.44, 95% CI 1.70–6.93; 500‐day washout: aHR 3.05, 95% CI 1.45–6.40). However, one exception was observed in the topiramate vs valproate comparison using a 180‐day washout period among patients with epilepsy, where the association became statistically non‐significant (aHR 2.00, 95% CI .94–4.26), although the point estimate remained elevated. Regarding the potential overlap of epilepsy and migraine at baseline, overall findings remained consistent with the primary results (Figure S4A,B), with aHRs continuing to show significantly elevated risks for both comparisons (vs valproate: aHR 3.70, 95% CI 1.46–9.36; vs lamotrigine: aHR 4.83, 95% CI 2.22–10.50). Stratified estimates demonstrated that the association was most pronounced in the epilepsy‐only group (vs valproate: aHR 3.49, 95% CI 1.55–7.83; vs lamotrigine: aHR 9.67, 95% CI 3.86–24.26), whereas results for the migraine‐only and epilepsy‐migraine groups were not statistically significant. Finally, patterns consistent with the primary results were also observed when the two‐prescription requirement was relaxed to include single‐prescription users, as shown in Figure S5A,B.

### 3.4 Post‐hoc analysis

In dose‐restricted analyses (Figure S6A,B), excluding the lowest 25% of topiramate dose users resulted in a higher risk of glaucoma compared with both valproate (aHR 3.33, 95% CI 1.42–7.83) and lamotrigine (aHR 4.86, 95% CI 2.32–10.17). When excluding the lowest 50% of topiramate dose users (Figure S7A,B), the magnitude of aHR increased further for each comparator, rising to 9.13 (95% CI 3.02–27.58) vs valproate and 13.04 (95% CI 5.13–33.16) vs lamotrigine. Subgroup patterns across sex, age, and indication were similar to those observed in the main analyses but with a higher magnitude.

The interaction of sex and indication was statistically significant in the comparison with valproate (*p* < .001) as the comparator but not significant in that with lamotrigine (*p* = .51) as the comparator. In Figure S8A,B, the increased glaucoma risk associated with topiramate was concentrated among women with epilepsy, who showed significantly elevated aHRs in both comparisons (vs valproate: 3.00, 95% CI 1.08–8.31; vs lamotrigine: 8.02, 95% CI 3.11–20.71). Women with migraine demonstrated a significant association only when topiramate was compared with valproate (vs valproate: aHR 3.95, 95% CI 1.11–14.03; vs lamotrigine: aHR 1.01, 95% CI .41–2.48), whereas no increased risk was observed among men with epilepsy or men with migraine in either comparator group.

After incorporating baseline estrogen use into IPTW‐weighted models (Figure S9A,B), the increased glaucoma risk associated with topiramate remained concentrated among women (vs valproate: aHR 5.30, 95% CI 1.47–19.06; vs lamotrigine: aHR 5.74, 95% CI 2.38–13.81), especially among those 18–49 years of age (vs valproate: aHR 5.53, 95% CI 2.06–14.83; vs lamotrigine: aHR 8.69, 95% CI 2.88–26.23), in both comparison groups. Stratification by baseline estrogen use showed that elevated risks persisted among estrogen non‐users (vs valproate: aHR 2.56, 95% CI 1.08–6.06; vs lamotrigine: aHR 3.61, 95% CI 1.78–7.35), whereas estimates among estrogen users were null due to very small sample size.

## 4 DISCUSSION

This study investigated the risk of glaucoma associated with topiramate use compared to valproate and lamotrigine in patients treated for epilepsy or migraine. Our findings demonstrated a significantly elevated glaucoma risk among topiramate users compared to both comparator groups. The cumulative risk among topiramate users rose sharply within the first few months after treatment initiation. Notably, the increased risk was most pronounced among female patients and those prescribed topiramate for epilepsy, whereas no significantly elevated risk was observed in male patients or in migraine patients.

Our study substantiates and comprehensively evaluates the association between topiramate and an increased risk of glaucoma. Specifically, although the nested case–control study by Symes et al.^10^ found a glaucoma rate ratio of 5.30 among topiramate users younger than 50 years compared to non‐users, neither theirs nor other earlier studies^8^, ^9^ conducted any additional subgroup analyses, such as exploring risk variations by sex or indication. Our study shows that female or epilepsy patients had a substantially higher risk, whereas male patients and those using topiramate for migraine treatment did not exhibit significant risk increases. This subgroup analysis offers clinically actionable insights into identifying high‐risk groups for more targeted monitoring. When compared to the cohort study conducted in Taiwan,^8^ our findings of one‐year follow‐up similarly indicate an overall elevated risk but with a greater magnitude. Moreover, although the cohort study of Ho et al.^8^ reported a 7.41‐fold increased risk with a wide CI (95% CI 2.45–22.46) in the first month and a sharp decline to 1.71 (95% CI 1.06–2.77) in a one‐year follow‐up compared to non‐users, we found that the cumulative risk associated with topiramate began to rise sharply at around Day 200 after drug initiation when compared to valproate and lamotrigine. These differences may be explained by the following factors. First, they did not exclude topiramate users who were also receiving other ASMs, which may confound the association, since some ASMs like zonisamide could independently influence glaucoma risk.^21^ Second, they did not report the average or median follow‐up duration and cumulative risk patterns, making their results not directly comparable with ours. Third, their one‐month estimates were based on a small number of glaucoma events with wide CIs, which increases the potential for statistical instability and sampling error. Fourth, our results are likely to reflect reduced indication bias and the focus on relative risks among ASM users, potentially capturing chronic rather than solely acute mechanisms. Finally, Ho et al. utilized data from 2001 to 2007, a period when case reports and case series of topiramate's ocular side effects were emerging, with the FDA issuing a black box warning for acute angle‐closure glaucoma in 2004.^22^ In contrast, our study extends up to September 2023, by which time warnings about the ocular side effect of topiramate were well established. This may have influenced prescribing practices, enhanced early ophthalmologic monitoring, or avoided use in high‐risk patients, thereby reducing acute early events and shifting the observed risk toward later‐onset patterns.

The higher glaucoma risk with topiramate in female patients or epilepsy patients may be attributable to several factors. The observed higher risk of glaucoma among female patients may be related to anatomic factors that could amplify susceptibility to topiramate‐induced fluid shifts.^23^ In addition, hormonal influences, including estrogen‐related changes in aqueous humor dynamics,^23^ may interact with the pharmacologic actions of ASMs to modify glaucoma susceptibility. Although prior studies suggest that estrogen may lower intraocular pressure,^24^ the elevated glaucoma risk associated with topiramate in our study persisted among women who had never used exogenous estrogen and varied in magnitude depending on the comparison ASM. The increased risk was primarily observed in women ages 18–49 years, whereas no significant association was found among women older than 50 years, who generally have lower endogenous estrogen levels. These findings suggest that although hormonal factors may contribute to sex differences in glaucoma susceptibility, they do not suffice to explain the topiramate‐associated risk observed in our study. Regarding indication‐specific differences, epilepsy patients on topiramate exhibited a markedly higher risk compared to those on valproate or lamotrigine. In our cohort, patients with epilepsy also received higher average daily dose of topiramate than migraine patients, which could amplify the potential mechanism of topiramate‐inducing complications. This possibility is further supported by our post hoc dose‐restricted analyses, in which excluding the lowest 25% and 50% of topiramate dose users led to progressively higher aHRs for both comparator groups. These findings together suggest that higher topiramate exposure may partly explain the elevated risk among epilepsy patients. Conversely, the absence of elevated risk in migraine patients might stem from lower doses typically used for prophylaxis, reducing the likelihood of threshold‐dependent adverse effects. Notably, several case reports have described glaucoma occurring in migraine patients after dose escalation of topiramate, suggesting that the risk may emerge when higher doses are reached.^25^, ^26^, ^27^ Future research should explore dose–response relationships and genetic factors, such as variations in carbonic anhydrase expression, to refine these explanations.

A key strength of our study over the existing literature is that the active‐comparator, new‐user cohort design effectively mitigates key biases inherent in prior observational studies, such as indication bias and prevalent user bias, by comparing topiramate initiators to those starting valproate or lamotrigine. This approach provides more clinically relevant insights for treatment selection among viable ASM options, unlike comparisons to non‐users in previous research, which are oftentimes unrealistic. However, several limitations should be acknowledged. First, as an observational study using electronic health records, residual confounding may persist despite comprehensive covariate adjustment, particularly for unmeasured factors such as family history of glaucoma and baseline ocular parameters such as axial length, intraocular pressure, and refraction status; we addressed this to the greatest extent by incorporating a wide range of established confounders, including demographics (age, sex, race), lifestyle factors (smoking, alcoholism), comorbidities (diabetes, hypertension, bipolar disorder, cataract), and concurrent medications (steroids, anticholinergics, antihistamines, antidepressants), selected based on their known associations with glaucoma risk and treatment assignment. Second, misclassification of exposures and outcomes is possible. For instance, glaucoma ascertainment relied on recorded diagnoses, procedures, or anti‐glaucoma prescriptions, which may underreport mild or asymptomatic cases not prompting primary care intervention. Drug discontinuation was estimated based on prescription data, potentially overlooking adherence variations. We mitigated this by using validated Read Codes and ICD‐10‐CM for diagnoses, supplementing diabetes and hypertension ascertainment with prescription records, requiring at least two prescriptions to define new users, and excluding patients with prior glaucoma history or anti‐glaucoma medications to minimize baseline misclassification. Third, subgroup analyses, although informative, were based on small event numbers in some strata (e.g., pediatrics or males), leading to wide CIs and reduced precision. Finally, our UK‐based cohort may limit generalizability to other populations, particularly those with higher proportions of ethnic groups prone to angle‐closure glaucoma (e.g., Asian populations), although we included race as a covariate in PS estimation to account for potential ethnic variations within the cohort.

Our findings have several direct implications for clinical practice. First, the markedly higher glaucoma risk in women initiating topiramate suggests that sex‐specific risk should be considered when selecting antiseizure therapy. Based on our effect estimates, avoiding topiramate could prevent approximately 25 glaucoma cases per 1000 female patients in the first treatment year compared with valproate. Similarly, the elevated risk in epilepsy suggests an indication‐specific effect: avoiding topiramate could prevent 26 cases per 1000 epilepsy patients vs valproate. In female patients with epilepsy who are at the intersection of both high‐risk groups, the absolute number of preventable cases is likely to be even greater. For those in whom topiramate remains the preferred option, baseline and periodic ophthalmologic evaluations during the first year may be warranted to enable early detection and intervention. Second, both lamotrigine and valproate emerged as effective comparators with substantially lower glaucoma risk, offering safer alternatives for epilepsy treatment from an ocular perspective. However, their own adverse‐effect profiles, such as teratogenicity and male fertility with valproate,^28^, ^29^ necessitate individualized benefit–risk assessment for each patient. Third, as sex‐specific differences in topiramate‐associated glaucoma risk have not been reported previously, these findings suggest that product labeling may need to be updated, particularly for epilepsy treatment. Finally, although we did not observe a statistically significant excess glaucoma risk among migraine patients at typical prophylactic doses, our study was not powered to rule out small to moderate effects, particularly at higher doses. Accordingly, clinicians may not need routine glaucoma monitoring in most migraine patients on topiramate, but a lower threshold for ophthalmologic assessment may be beneficial for those taking high‐dose regimens or with other glaucoma risk factors. In addition, topiramate use for migraine should be weighed carefully when prescribed to women of reproductive age due to its known teratogenic risk.^30^, ^31^

## 5 CONCLUSIONS

In conclusion, topiramate use was associated with a significantly higher risk of glaucoma compared to valproate and lamotrigine, particularly among female patients and those treated for epilepsy. No significant risk was observed in migraine patients or male patients, indicating that risk profiles may vary based on sex and indication; further studies are needed to explore dose‐dependent effects and the underlying mechanisms.

## AUTHOR CONTRIBUTIONS

F.T.T.L. and C.W. conceived the idea and design of the study. C.W. performed data collection, extraction, and analysis. C.W. drafted the paper. All authors reviewed the final manuscript. All authors have read and approved the final submitted manuscript and agree to be accountable for the work.

## FUNDING INFORMATION

This research did not receive any specific grant from funding agencies in the public, commercial, or not‐for‐profit sectors.

## CONFLICT OF INTEREST STATEMENT

The authors declare no competing interests.

## ETHICS STATEMENT

The study was approved by the Scientific Review Committee of the IQVIA (25SRC004). We confirm that we have read the Journal's position on issues involved in ethical publication and affirm that this report is consistent with those guidelines.

## SOCIAL MEDIA AND ARTICLE PROMOTION

A team from Hong Kong led by @clairewei0830 identified increased glaucoma risks following topiramate use, particularly in women and epilepsy patients, in UK primary care data. @FranciscoTTLai1 @Esther_CSMPRHKU @CSMPR_HKUPharm.

## Supporting information


Data S1.

## Data Availability

The supporting data for this study are not publicly available owing to their proprietary nature. However, they can be provided upon reasonable request to the corresponding author, subject to compliance with applicable regulations and institutional policies.
