# Supplementary material for: Sex‐specific elevated incidence of glaucoma associated with topiramate versus valproate or lamotrigine in epilepsy, not migraine: A population‐based cohort study
Source: Epilepsia. 2026 Jan 10;67(4):1690–702. doi: 10.1002/epi.70087 (PMC13075613; doi:10.1002/epi.70087)

**SUPPLEMENTARY TABLES**

Table S1. List of diagnosis codes and medication codes

| Diagnosis or procedure | Read codes, Version 2 | International Classification of Diseases, Tenth Revision, Clinical Modification (ICD-10-CM) |
| --- | --- | --- |
| Epilepsy | F25, 1473, 667N, 1030, 1JA0, 667, 13Y9, 9N0r, 667K, 8BIF, 8Hlp, 8T0L, 90f, 667L, 90f4, 67AF, 6110, 67IJ0, 8IAi, 8IAg, 8IAh | G40 |
| Migraine | F26, 1474, 8B6N | G43 |
| Diabetes | 66A, 1434, C10F, C10E, 9NM0, 8H7f, 90L, 8HTE1 | E10-E14 |
| Hypertension | 14A2, G2, 662P0, 9N03 | I10-I15 |
| Bipolar disorder | E11, Eu31, Eu30 | F31 |
| Cataract | F46, 1483, 22E5, 8LC0, 2BT1, 8HTV, 2BT0, 726, 8H5H, 14NC | H25-H28 |
| Smoke | 137, H3101, ZV4D7 | Z72.0, Z87.891, F17.2 |
| Alcoholism | E23, 1462, Eu102, ZV113, 8H35 | F10 |
| Glaucoma | F45, 1482, 1JF, 66T1, F4042, 8HTW, 7259 | H40-H42 |
| Laser trabeculoplasty | 72560 | - |
| Trabeculectomy | 72550, 72556, 72587 | - |
| Laser peripheral iridotomy | 72571, 72572 | - |
| Cyclophotocoagulation | 72603, 7275 | - |
| Operations on anterior or posterior chamber | F4455, 726 | - |
|  | | |
| Medication | Anatomical Therapeutic Chemical (ATC) | |
| Antiepileptics | N03A | |
| Antidepressants | N06A | |
| Anticholinergics | N04A | |
| Antihistamines | R06 | |
| Antihypertensives | C02 | |
| Antidiabetics | A10 | |
| Estrogen | G03A, G03C and G03F | |

**SUPPLEMENTARY FIGURES**

Figure S1a. Comparison of standardized mean differences across crude and weighted covariates (topiramate and valproate cohort)


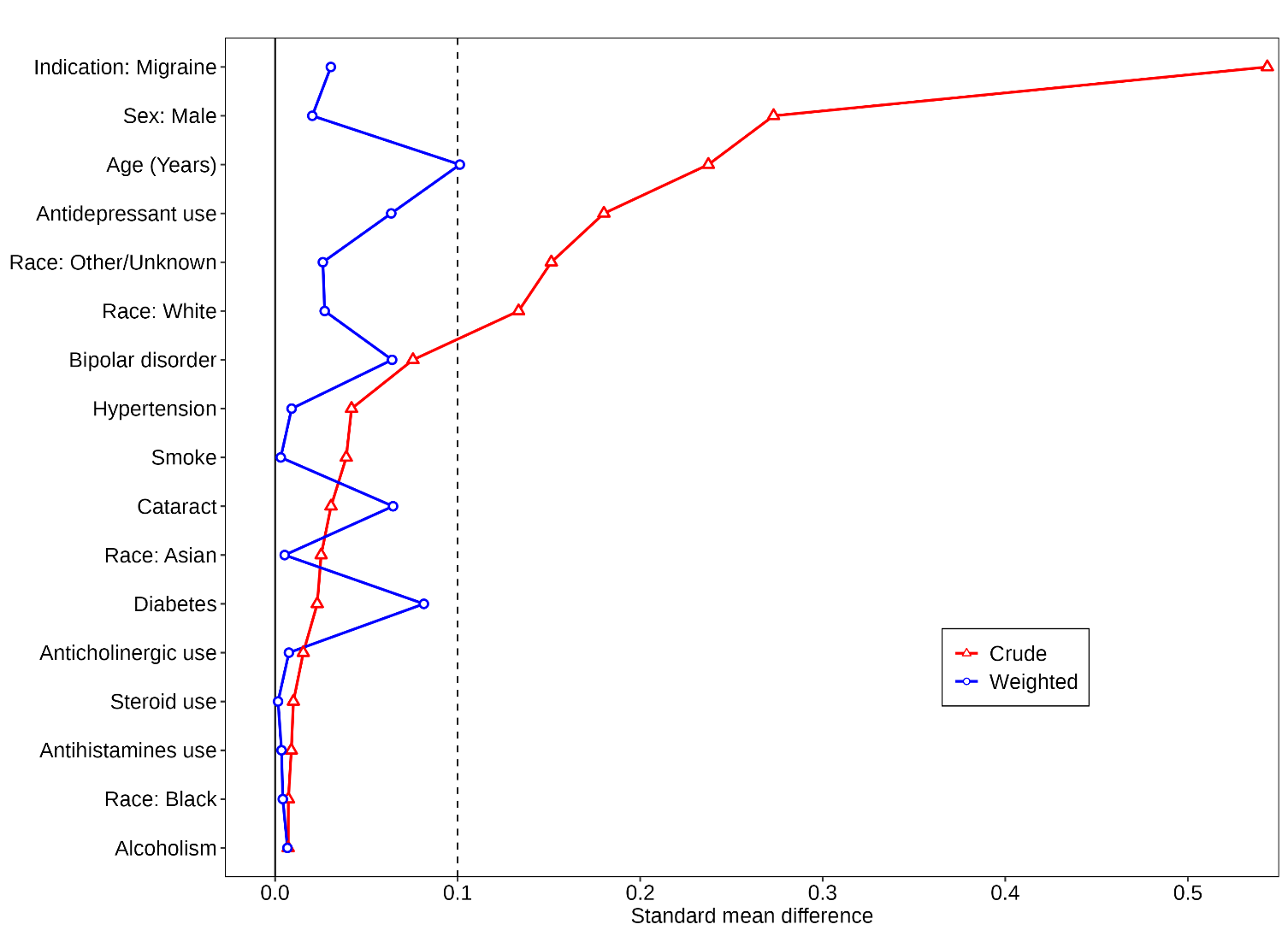


Figure S1b. Comparison of standardized mean differences across crude and weighted covariates (topiramate and lamotrigine cohort)


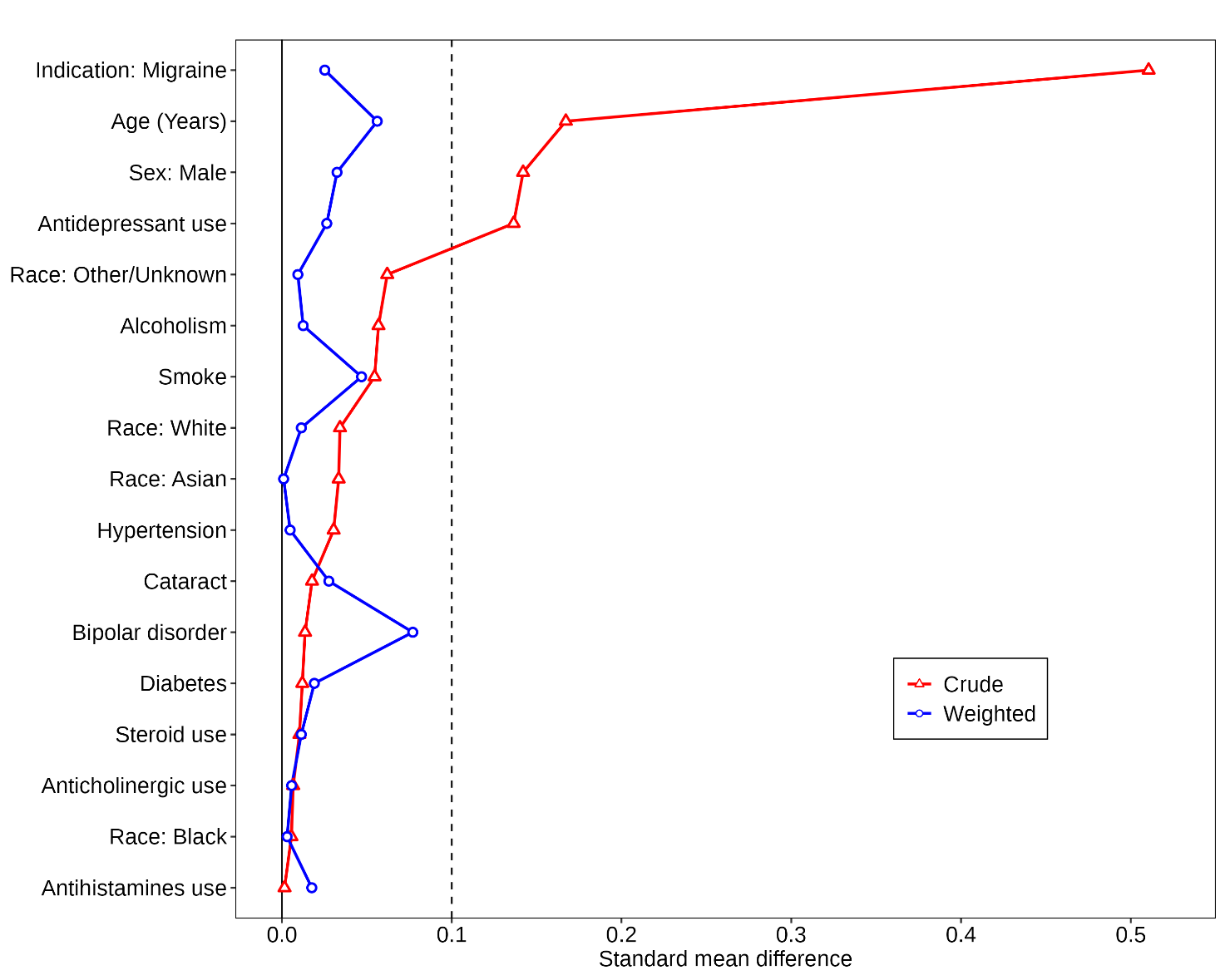


Figure S2a. Forest plot of main and subgroup results (topiramate and valproate cohort with a 180-day washout period)


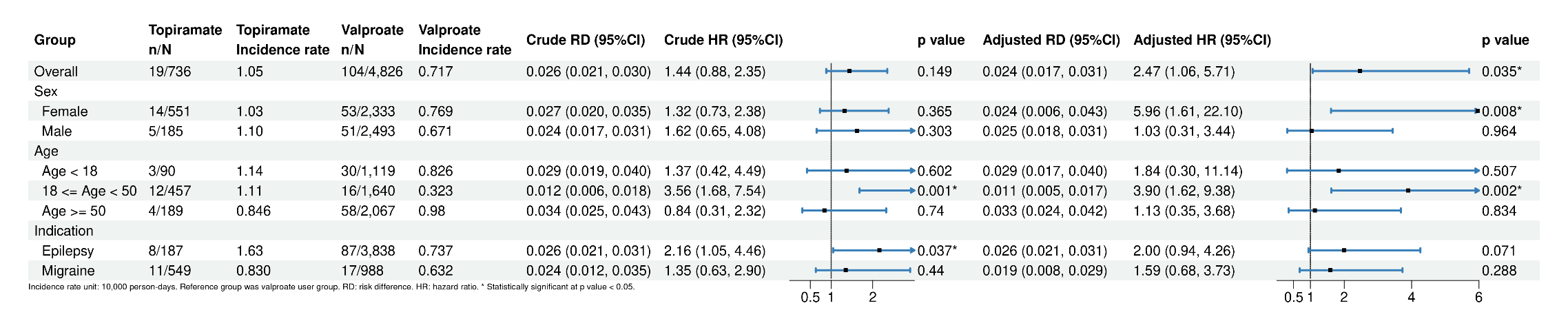


Figure S2b. Forest plot of main and subgroup results (topiramate and lamotrigine cohort with a 180-day washout period)


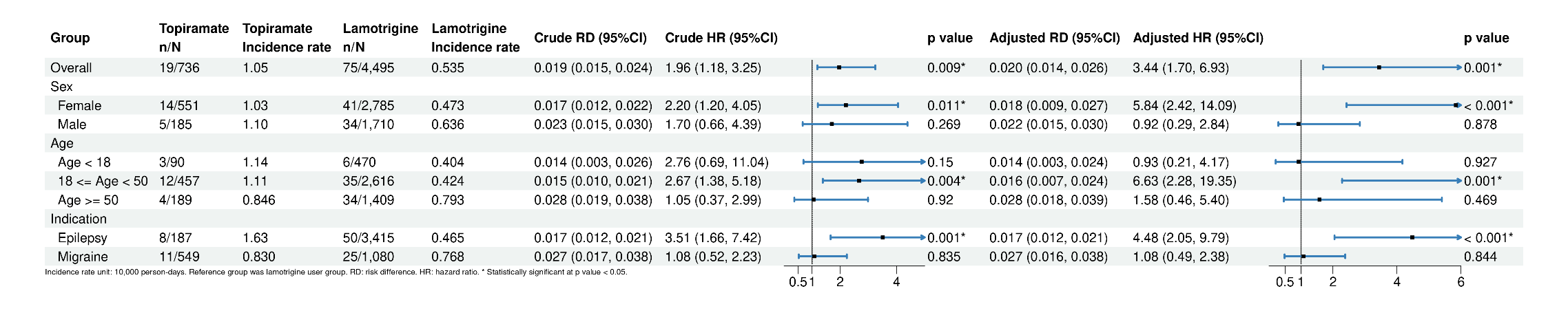


Figure S3a. Forest plot of main and subgroup results (topiramate and valproate cohort with a 500-day washout period)


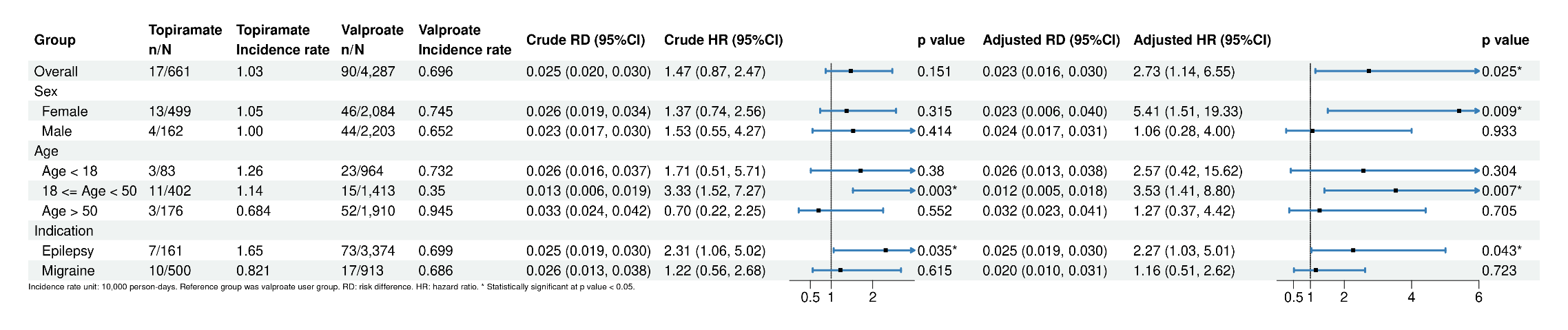


Figure S3b. Forest plot of main and subgroup results (topiramate and lamotrigine cohort with a 500-day washout period)


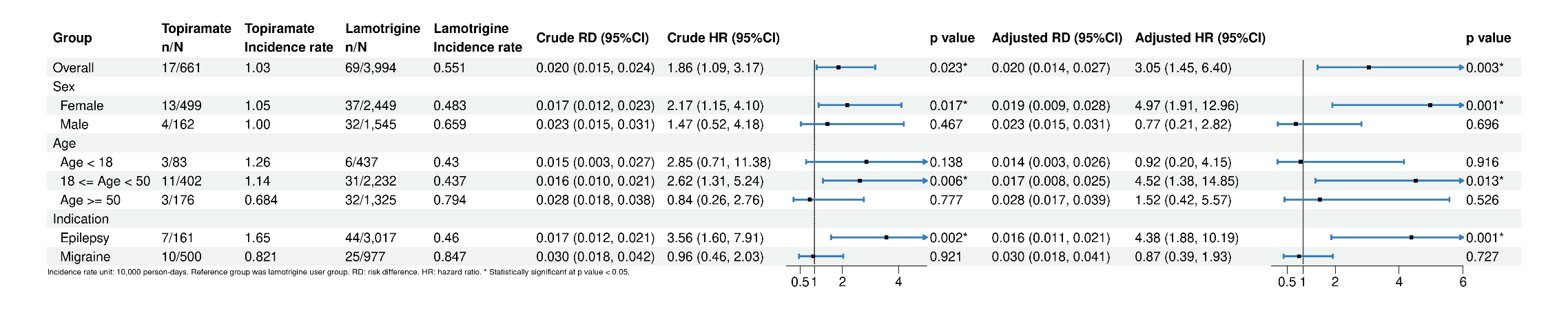


Figure S4a. Forest plot of main and subgroup results (topiramate and valproate cohort with indication redefinition)


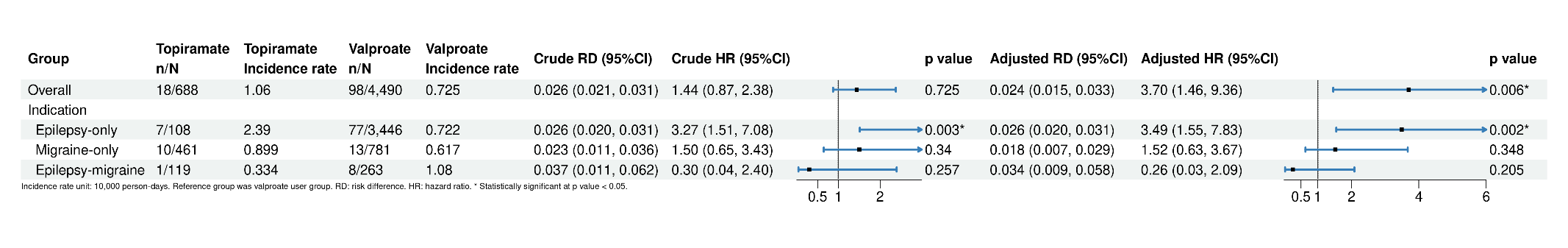


Figure S4b. Forest plot of main and subgroup results (topiramate and lamotrigine cohort with indication redefinition)


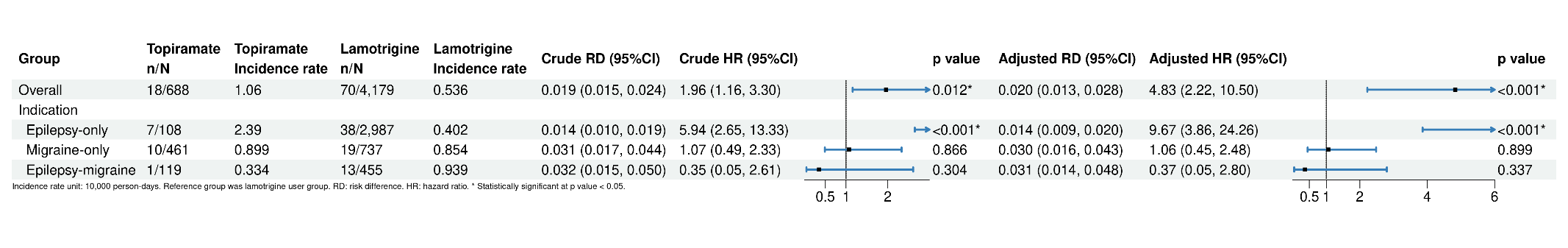


Figure S5a. Forest plot of main and subgroup results (topiramate and valproate cohort with at least one prescription)


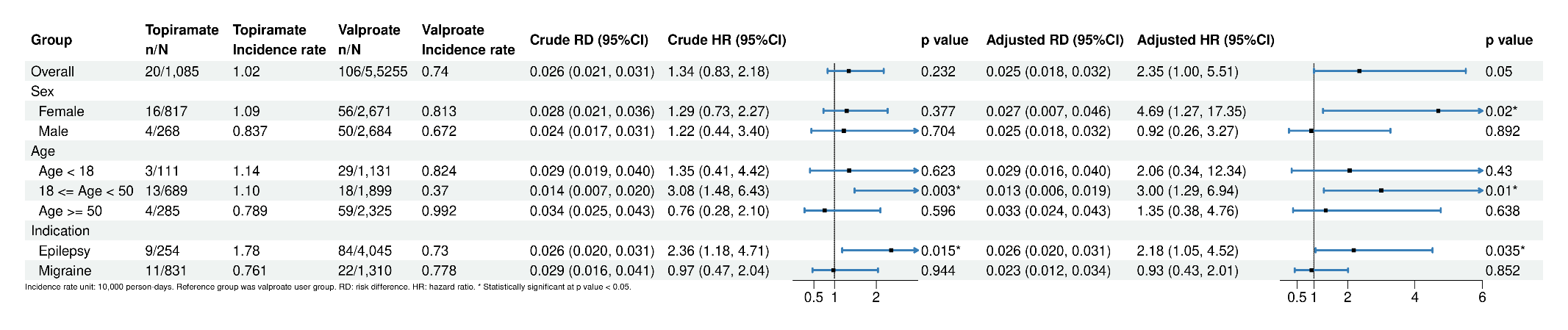


Figure S5b. Forest plot of main and subgroup results (topiramate and lamotrigine cohort with at least one prescription)


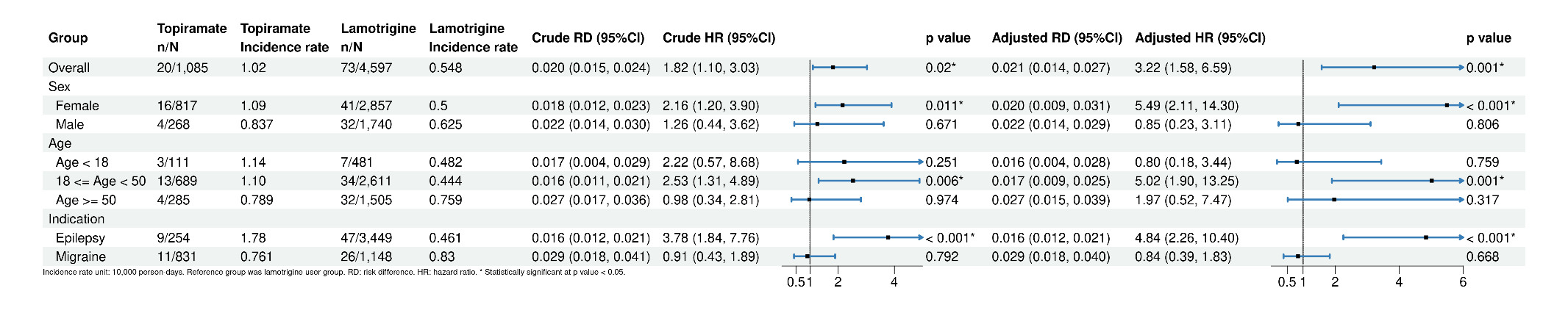


Figure S6a. Forest plot of main and subgroup results (topiramate and valproate cohort with excluding the 25% lowest average daily dosage topiramate users)


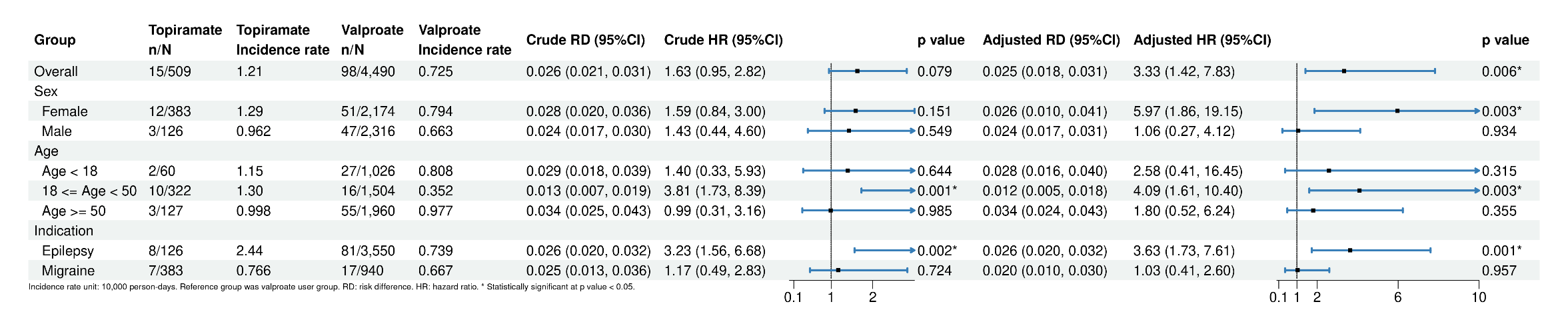


Figure S6b. Forest plot of main and subgroup results (topiramate and lamotrigine cohort with excluding the 25% lowest average daily dosage topiramate users)


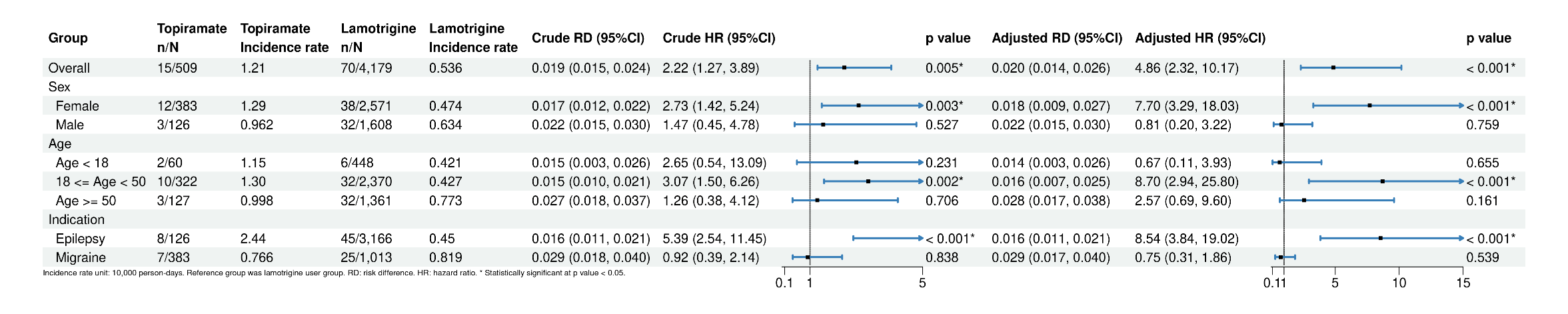


Figure S7a. Forest plot of main and subgroup results (topiramate and valproate cohort with excluding the 50% lowest average daily dosage topiramate users)


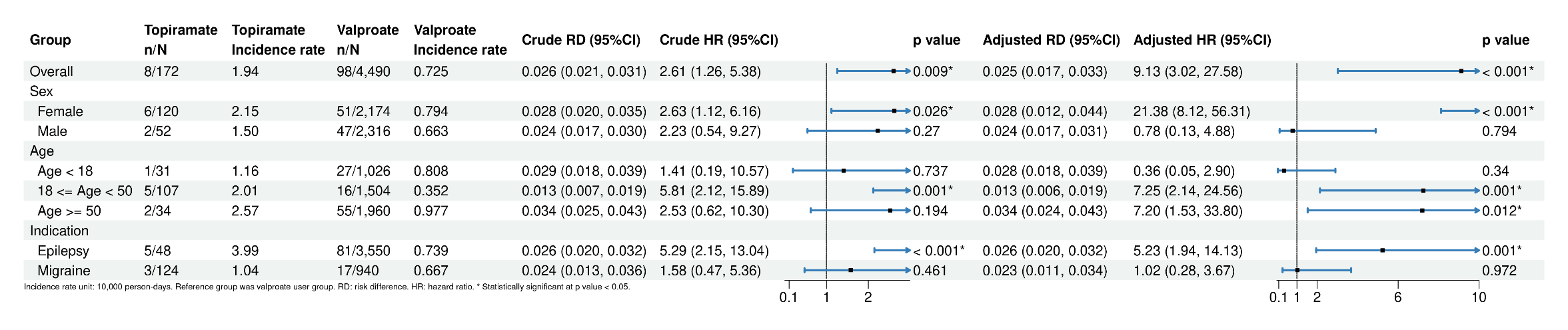


Figure S7b. Forest plot of main and subgroup results (topiramate and lamotrigine cohort with excluding the 50% lowest average daily dosage topiramate users)


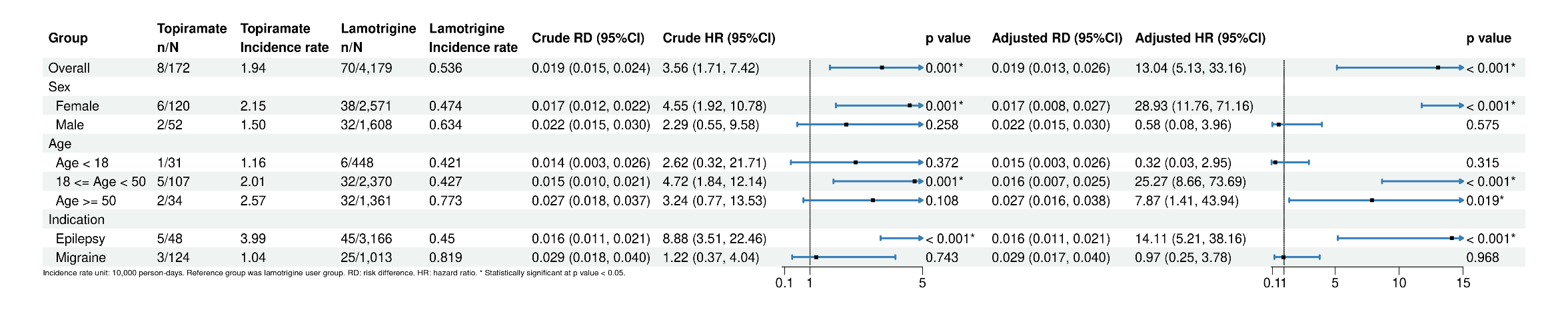


Figure S8a. Forest plot of main and subgroup results (topiramate and valproate cohort with stratification by sex and indication)


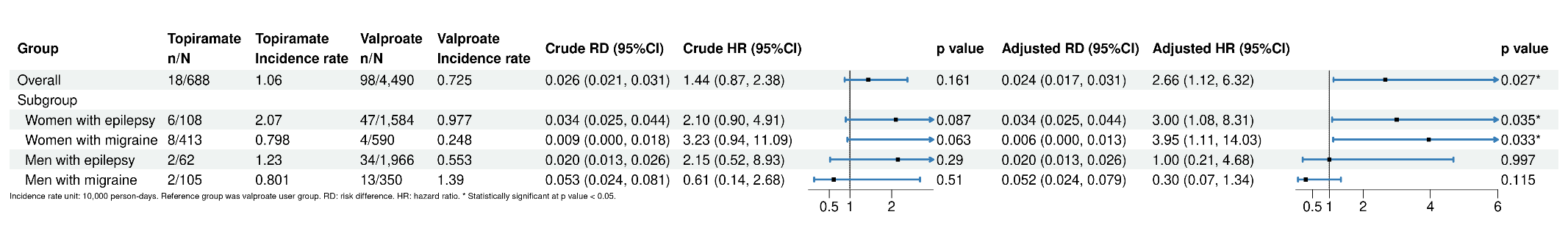


Figure S8b. Forest plot of main and subgroup results (topiramate and lamotrigine cohort with stratification by sex and indication)


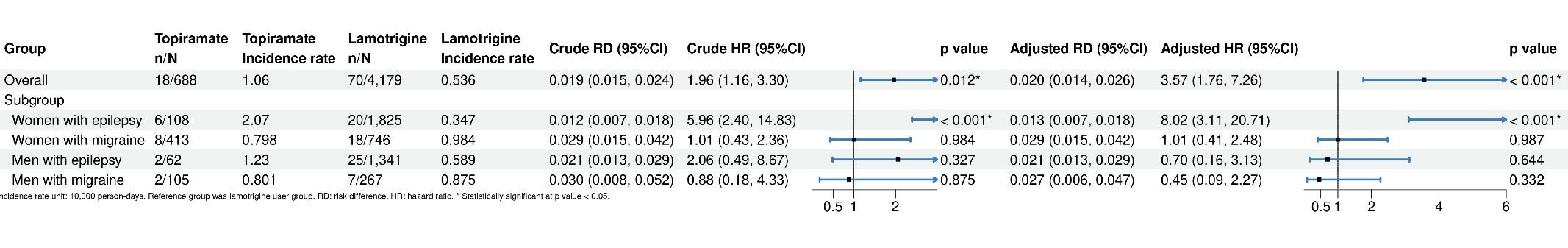


Figure S9a. Forest plot of main and subgroup results (topiramate and valproate cohort with estrogen use at baseline)


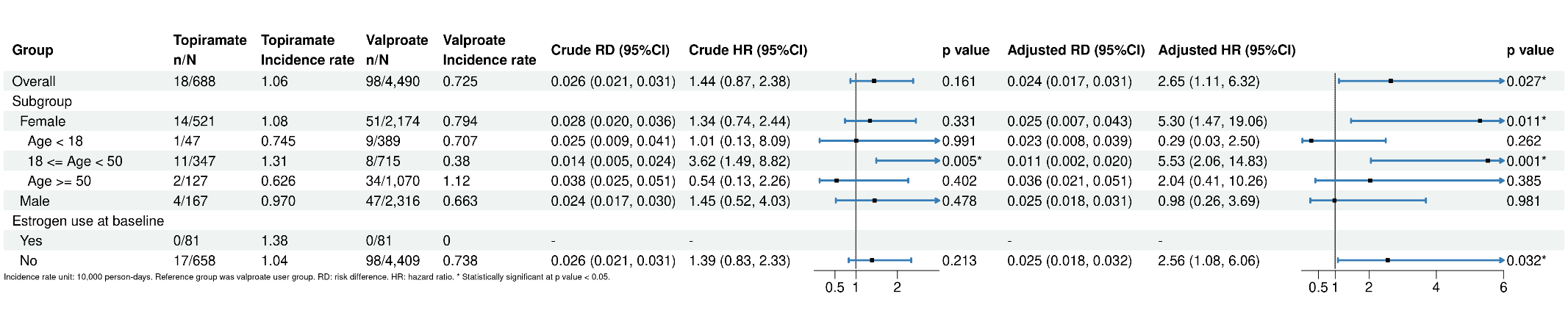


Figure S9b. Forest plot of main and subgroup results (topiramate and lamotrigine cohort with estrogen use at baseline)


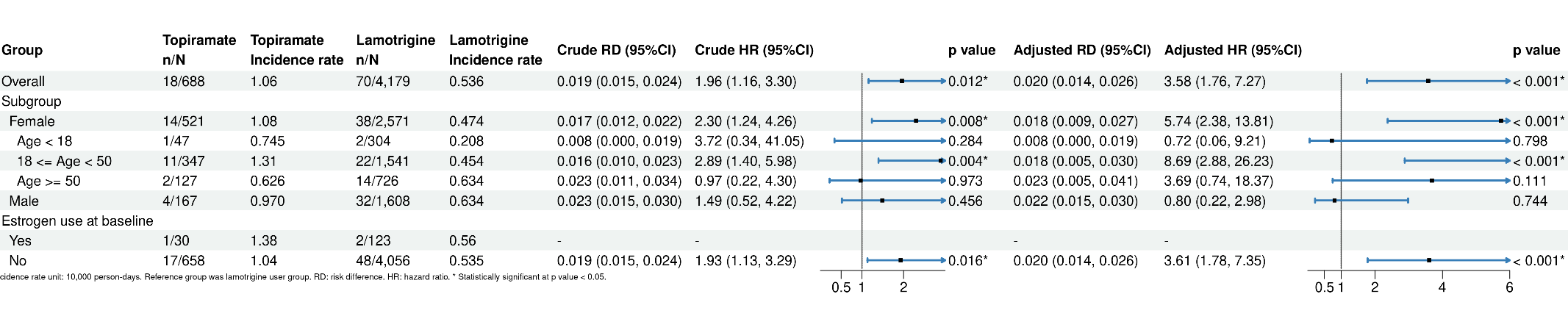

Supplement: Supplementary file 1 — Data S1. [file EPI-67-1690-s001.docx]
